# Texas Homœopathic Medical Society

**Published:** 1888-06

**Authors:** 


### TEXAS HOMŒOPATHIC MEDICAL SOCIETY.

The fifth annual session of the Texas Homœopathic Society met at Dallas, May 1st. The attendance was small, but those present showed they were alive and active. The meeting was called to order at eleven A. M. by the President, S. W. Cohen, M. D., of Waco.

Rev. C. I. Scofield, of the First Congregational Church, of Dallas, opened the convention with prayer.

The roll-call by the Secretary and numerous letters and telegrams of regret received from various portions of the State revealed the fact that only a fraction of the members was to be present. However, those who were present strove to make up the deficiencies, and proceeded to business in an earnest manner.

An address of welcome, from Dr. W. S. Lee, of Dallas, was read by the Secretary, in which was detailed briefly the pioneer work of Homœopathy in Dallas.

The Society returned a resolution of thanks to Dr. Lee, which embodied expressions of the kindliest, fraternal feeling for the Doctor as the pioneer in this portion of the State, and sympathy was extended him in his present inactive condition, trusting that he may be speedily restored to his wonted health and usefulness.

The day was occupied chiefly in routine business of considerable interest to physicians.

President Cohen presented a series of recommendations to the Association for their consideration, one of chief interest to the profession and laity referring to medical legislation.

In the afternoon the President delivered his annual address, which was received with marked interest by the members present, and a vote of thanks was tendered him.

The work of the bureaus was taken up, and interesting papers were read by various members on the following subjects: "Materia Medica," "Theory and Practice," "Obstetrics," "Gynæcology," "Pædology," "Surgery," and "Institutes of Homœopathy."

The officers elected were as follows: President, F. Hines, M. D., Corsicana; First Vice-President, Dr. Edwards, of Blanco; Second Vice-President, G. W. Sherbino, M. D., Abilene; Secretary, G. G. Clifford, of San Antonio; Treasurer, W. F. Thacker, M. D., Dallas. The Society will hold its next session at Fort Worth, beginning on the second Tuesday of May, 1889.

